# Supplementary material for: Chromothripsis during telomere crisis is independent of NHEJ, and consistent with a replicative origin
Source: Genome Res. 2019 May;29(5):737–49. doi: 10.1101/gr.240705.118 (PMC6499312; doi:10.1101/gr.240705.118)
Supplement: Supplemental Material [file supp_gr.240705.118_Supplemental_file_1.zip › contigs/annotated_contigs/DB53/contig.2.DB53_length_400_mean_cov_7.4675.docx]

**DB53_length_400_mean_cov_7.4675**

```
CATGGTACTGCTACAAAAACAGACACATAGACCAACGGAACAGAATAGCGAATCAAGATATAAGGCTGCAGCCTGGGTGCAGTGGCTCA
 >chr1:243114612-243114960 + E=2e-195
CACCTGTAATCCCAGCACTTTGGGAGGCTGAGGTGGGTGGATCACCTGAGGTTGGGAGTTCAAAACCAGTCTGACCAACTTGGAGAAAC

CCCATCTCTACTAAAAATACAAAATTAGCTGGGCGTTGTAGTGCATGCCTGTAATCCCAGCTACTCAGGAGGCTGAGGCAGGAGAATCG

CTTGAACCCAGGAGGCAGAGGTTGCGGTGAGTGGAGATTGCACCATTACACTCCAGCCTGGGCAACAAGAGGAA|AAAGAAG|AAAGAA
                                                                           >chr20:46540
AGAAAGAAAGAAAGAAAGAAAGAAAGAAAGAAAGAAAGAAAGAAAG208-46540267 - E=5e-24
```
